# When a Headache Is More than the Flu: A Case Report

**DOI:** 10.5811/cpcem2022.6.56491

**Published:** 2022-08-08

**Authors:** Abigail E. Russ, Amber M. Morse, David M. Spiro

**Affiliations:** University of Arkansas for Medical Sciences, Arkansas Children’s Hospital, Department of Pediatric Emergency Medicine, Little Rock, Arkansas

**Keywords:** Pott’s puffy tumor, frontal bone osteomyelitis, influenza, case report, sinusitis

## Abstract

**Introduction:**

When influenza (flu) season arrives, it is easy for emergency department clinicians to anchor on the diagnosis of flu, sending patients on their way with or without anti-influenza medication. It is important not to miss the outlier – the patient who seems to have typical symptoms of influenza but with certain subtleties that should make one consider expanding the differential diagnosis.

**Case Report:**

We describe an 11-year-old previously healthy male who presented with eight days of fever, myalgias, cough, congestion, and headache in the context of positive influenza exposure. The length and severity of his symptoms prompted laboratory and imaging investigation. He was positive for influenza type B with elevated inflammatory markers but otherwise normal laboratory workup and normal chest radiograph. He complained of a headache and was given fluids and antipyretics, and was admitted for overnight observation. He specifically did not have any forehead swelling. The next day during his inpatient stay he developed right frontal forehead edema and appeared ill. He was taken for a sinus computed tomography, which showed changes consistent with frontal bone osteomyelitis. Even after drainage by neurosurgery and otolaryngology, the patient subsequently developed repeat abscesses and ultimately a superior sagittal sinus thrombosis.

**Conclusion:**

Other sources of infection should be considered in patients who have flu-like symptoms that last longer than expected, present with focality, or appear ill.

## INTRODUCTION

When influenza (flu) season arrives, it is easy for clinicians in the emergency department (ED) to anchor on the diagnosis of flu, sending patients on their way with or without anti-influenza medication. It is important not to miss the outlier – the patient who seems to have typical symptoms of influenza but with certain subtleties that should make one consider expanding the differential diagnosis.

## CASE REPORT

A previously healthy 11-year-old male presented to an urban pediatric ED complaining of fever, rhinorrhea, congestion, cough, myalgias, and headache. His mother, father, and brothers had all been diagnosed with flu over the prior several days and had recovered within three to four days. The patient had a negative influenza test at an urgent care center. He had not received the current flu vaccine but otherwise was fully vaccinated. He had daily fevers, up to 104° Fahrenheit, for eight days prior to evaluation in the ED, with worsening rhinorrhea and an intermittent frontal headache. He had received antipyretics but no other medications. His physical exam was significant for a pale, thin, and ill-appearing male. He had tenderness to palpation of his right and middle forehead without overlying swelling or discoloration. He had full range of motion of his neck, dry mucus membranes, tonsil stones present in the oropharynx, normal heart and lung findings, and no neurological abnormalities, specifically no ataxia, meningismus, peripheral nerve deficits, or cranial nerve deficits.

Our differential diagnosis at the time included an atypical presentation of influenza, mononucleosis infection, sinusitis, migraine, Kawasaki disease, dehydration, and sepsis. His neurological exam did not make us suspicious for meningitis. He had no joint or bony pain or swelling that led us to include a joint infection or osteomyelitis in our differential diagnosis. He was given an antipyretic for fever and a fluid bolus for borderline tachycardia and dehydration. Laboratory workup was significant for a negative rapid influenza test but a positive respiratory pathogen polymerase chain reaction test for influenza B. His C-reactive protein was elevated at 102.0 milligrams per deciliter (mg/dL) (reference range: 0.0–9.9 mg/dL). Blood and urine cultures were obtained. A complete blood count, comprehensive metabolic panel, urinalysis, rapid streptococcus test, ferritin, anti-streptolysin O titer, and Monospot test were all within normal limits. A chest radiograph showed no abnormalities. An electrocardiogram showed normal sinus rhythm. Given his length of fever, ill appearance and elevated inflammatory markers, he was admitted for overnight observation and intravenous fluids. Antibiotics were not started secondary to a lack of nidus for bacterial infection.

**Image 1:**
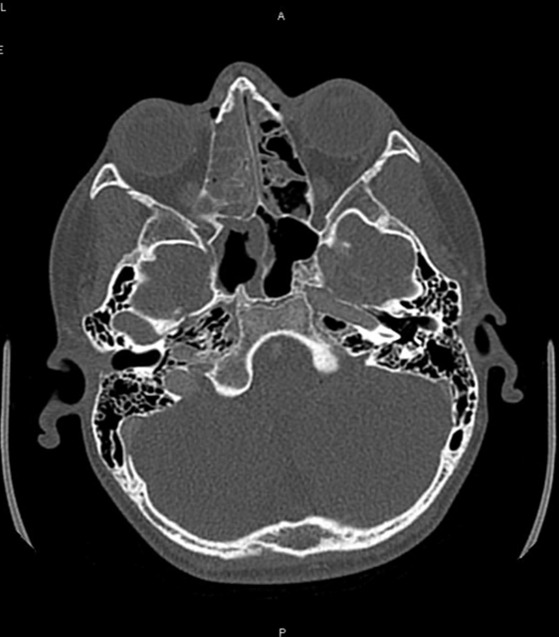
Computed tomography of our patient showing right ethmoid (white arrow) and maxillary (white arrowhead) sinusitis with enhancement demonstrating abscess formation (arrow).

He continued to be febrile overnight. The next morning, he was febrile, tachycardic, and more ill-appearing with headache and pronounced swelling over the right-middle forehead. A sinus computed tomography with contrast (Image 1) was obtained and demonstrated a midline frontal empyema displacing and narrowing the superior sagittal sinus over a segment measuring nine centimeters (cm). The empyema measured 1 cm in thickness. A subperiosteal abscess was seen in the left frontal region with surrounding phlegmonous changes of the scalp. Although no obvious lytic lesions were seen within the calvarium, the presence of subperiosteal abscess and intracranial empyema was concerning for frontal bone osteomyelitis, also described in the literature as Pott’s puffy tumor. Additionally, acute sinus disease involving bilateral frontal, right ethmoid, right sphenoid, and right maxillary sinus was seen.

**CPC-EM Capsule**

What do we already know about this clinical entity?

*We know frontal bone osteomyelitis (i.e. Pott’s puffy tumor) is a rare occurrence in children and is usually a complication of sinusitis, trauma, acute otitis media, or insect stings*.

What makes this presentation of disease reportable?

*Very rarely has frontal bone osteomyelitis occurred in conjunction with influenza infection*.

What is the major learning point?

*The most common symptoms of frontal bone osteomyelitis are headache and fever, which are also two common influenza symptoms. It is important to consider other diagnoses in a patient with viral disease or illness*.

How might this improve emergency medicine practice?

*Consideration of other disease processes will lead to prompt recognition and management of a serious disease that can have dire neurological complications if left untreated*.

The patient was taken to the operating room (OR) by both neurosurgery and otolaryngology for a right frontal craniotomy for evacuation of the abscesses and a functional endoscopic sinus surgery with septoplasty. After surgery, he was taken to the pediatric intensive care unit (PICU) for overnight monitoring and broad-spectrum antibiotics were initiated including ceftriaxone, vancomycin, and metronidazole. A brain magnetic resonance image (MRI) was obtained postoperatively that showed post-surgical changes, resolution of the abscesses, and no further evidence of osteomyelitis. Wound cultures from the intracranial abscesses grew *Streptococcus anginosus* that was sensitive to ceftriaxone and metronidazole. He had a peripherally inserted central catheter placed and was discharged home after eight days of hospitalization on IV ceftriaxone and oral metronidazole for six weeks of outpatient antimicrobial therapy.

**Image 2:**
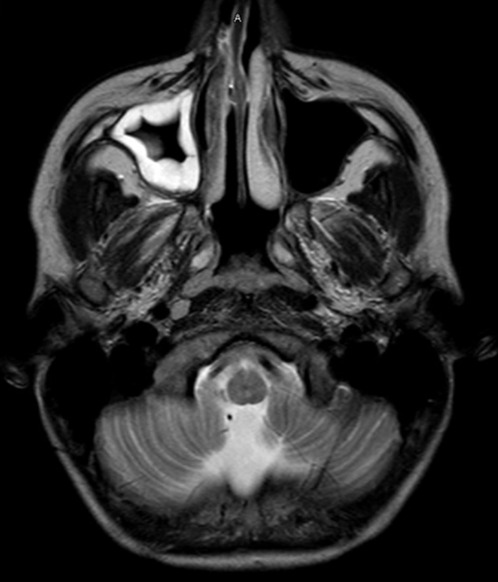

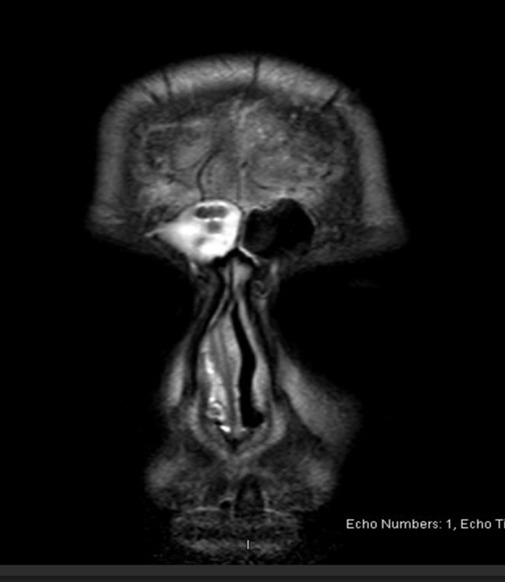
Brain magnetic resonance imaging with contrast of our patient. A) Demonstrates significant mucosal thickening of the maxillary sinus (arrow) consistent with recurrent sinusitis. B) Demonstrates return of right frontal osteomyelitis (arrow).

After discharge home, he returned to the ED two days later with slowed speech, difficulty finding his words, ataxia, and headache. A brain MRI with contrast was ordered and showed right frontal bone osteomyelitis with abscesses overlying the right frontal bone and an anterior superior sagittal sinus thrombosis (Image 2). He was taken to the OR by neurosurgery for drainage of the subcutaneous, epidural, and subperiosteal abscesses, and a subgaleal drain was placed. He was transferred postoperatively to the PICU for anticoagulation, anti-seizure prophylaxis, and monitoring. His wound cultures did not grow any bacteria or fungus. He was discharged home and continued ceftriaxone and metronidazole and enoxaparin injections. At his subsequent follow-up visits, he continued to have no further complications and has made a full neurological recovery.

## DISCUSSION

The description of Pott’s puffy tumor was first penned by Sir Percivall Pott in the mid-1700s.^1^ The “tumor” refers to the forehead edema that occurs as a reaction to the underlying frontal bony infection. The infection may be isolated to the bone (ie, osteomyelitis) or may create an abscess in the subcutaneous tissue or brain. The most common cause of the tumor is extension of sinusitis into the frontal bone; however, although rare, trauma, acute otitis media, and insect stings have also been implicated.^2^,^4^

The most common bacteria associated with this process is the *Streptococcus anginosus* group, which includes *Streptococcus anginosus, Streptococcus intermedius*, and *Streptococcus constellatus*.^2^ Polymicrobial infections have also been described in the literature, most commonly a combination of anaerobic bacteria and *Staphylococcus aureus*.^1^ *S. anginosus* has been implicated in infections throughout the body as it is a member of the body’s natural flora, primarily in the mouth and the gastrointestinal tract. *S. anginosus* can cause life-threatening head and neck abscesses as well as septic thrombi in the brain and must be taken seriously.^3^

The majority of patients reported throughout the literature over the last 40 years were teenagers who presented with headache, fever, and scalp swelling. Depending on the extent of the abscess, patients may present with neurological deficits such as aphasia, cranial nerve deficit, and hemiplegia, although these seem to be infrequent complications.^1^ Given the intimately adjoining anatomy of bone, dura, and vasculature of the frontal sinus, infection can spread quickly, invading the brain and not only causing abscesses, meningitis, and bacteremia but also septic thrombi, leading to microvascular damage, strokes, and other severe neurologic sequelae. As in our patient, septic thrombi can form and remain subclinical in nature, only diagnosed on angiography, thus emphasizing the importance of obtaining vascular imaging from the first diagnostic scan.

Antimicrobial management alone is inadequate for treatment of these intracranial abscesses. If no abscess is present, one could argue that antimicrobial management with close observation for abscess formation may be adequate. However, for definitive management, neurosurgical intervention is required. Some patients have required a bone flap and multiple visits to the OR, as seen in our patient.

The association between influenza and Pott’s puffy tumor has been described in one other case series by Foster et al in 2019. Their report describes six patients with head and neck infections with influenza co-infection. One of these patients was a 12-year-old male with influenza who on day four of illness developed left eye pain with left eye swelling and left forehead swelling. He was diagnosed with sinusitis, Pott’s puffy tumor, and orbital cellulitis. He was taken to the OR for endoscopic sinus surgery. Cultures grew *S anginosus* group, *Streptococcus mitis*, *Peptostreptococcus*, and *Prevotella*. He was treated with ceftriaxone and metronidazole for five weeks. This patient was readmitted two weeks after discharge for vomiting and postnasal drip and did not have any reported neurological dysfunction.^5^

## CONCLUSION

Although rare, given its potential for serious neurological complications, Pott’s puffy tumor should be considered in the differential diagnosis for all patients with headache, fever, facial pain, and/or facial swelling. These symptoms may be consistent with the diagnosis of influenza but should not lead a diagnostician down the path of excluding other diagnoses, as co-infection is possible. The most common cause of Pott’s puffy tumor is sinusitis, although the absence of sinusitis does not exclude the diagnosis. Influenza may predispose a patient to subsequent bacterial infections as seen in our patient. This case demonstrates the need for clinicians to consider other diagnoses in the context of a definitive diagnosis of influenza.
